# Beier, C., Messner, D., & Preuß, H.-J. (Hrsg.) (2020). *Globale Wanderungsbewegungen. Beiträge der internationalen Zusammenarbeit zum Umgang mit Flucht und Migration.* Wiesbaden: Springer VS. 346 S., ISBN: 978-3-658-28236-3, € 49,99.

**DOI:** 10.1007/s12399-021-00881-6

**Published:** 2021-12-21

**Authors:** Rainer Lenzen

**Affiliations:** Forschungsinstitut für Politische Wissenschaft und Europäische Fragen, Universität zu Köln, Gottfried-Keller-Str. 6, 50931 Köln, Deutschland

Die nun schon fast zwei Jahre andauernde Omnipräsenz von Covid-19 in Politik und Medien hat andere Themen in den Hintergrund gerückt, ohne dass diese an Bedeutung und Aktualität verloren hätten. Dazu zählen auf globaler Ebene etwa geopolitische sowie klimatische Entwicklungen oder eben das Thema Migrationsbewegungen, denen sich der von Christoph Beier, Dirk Messner und Hans-Joachim Preuß herausgegebene Sammelband *Globale Wanderungsbewegungen. Beiträge der internationalen Zusammenarbeit zum Umgang mit Flucht und Migration* widmet.

Der EU-Türkei-Aktionsplan, der einen vorläufigen Schlusspunkt unter die 2015er-/2016er-Migrationswelle in Richtung Europa bzw. Europäische Union gesetzt hatte, stellt eher eine Bekämpfung der Symptome denn der Ursachen dar. Dass das Ausmaß globaler Wanderungsbewegungen (weiterhin) eine der zentralen Herausforderungen des 21. Jahrhunderts ist und einer entsprechenden Aufmerksamkeit bedarf, macht der Band mehr als deutlich.

Der Sammelbänden oftmals inhärenten Problematik, die einzelnen Beiträge in eine stringente Argumentationslinie zu bringen, begegnen die Herausgeber mit dem originellen Konzept, dass „der Aufbau des Buches [.] den Wegen von Flüchtlingen und Migrant/innen [folgt]“ (S. 6). Auf diese Weise gelingt den Herausgeber eine umfassende und schlüssige Gesamtschau des facettenreichen Untersuchungsgegenstands globaler Wanderungsbewegungen, den der Sammelband aus verschiedenen Perspektiven beleuchtet. Den Rahmen dafür setzen die ersten beiden Kapitel. Darin geben die Herausgeber (Kap. 1) und Daniel Naujoks (Kap. 2) einen einführenden Überblick über die Gesamtthematik sowie aktuelle Entwicklungen in der internationalen Migration und in der Flüchtlingsproblematik.

David Benček, Matthias Lücke, Claas Schneiderheinze und Tobias Stöhr (Kap. 3) untersuchen die Auswirkungen regulärer und irregulärer Migration auf Herkunftsländer und Aufnahmeländer. Jan Schneider (Kap. 4) beschreibt, welche Potenziale die Zusammenarbeit von Akteuren der Ordnungs- und Migrationspolitik und Akteuren der Entwicklungspolitik bei der freiwilligen Rückkehr von Migrant*innen in ihre Herkunftsländer bietet. Martin Weiß und Stephanie Deubler (Kap. 5) evaluieren anhand des von der Deutschen Gesellschaft für Internationale Zusammenarbeit (GIZ) am Horn von Afrika umgesetzten Projektes Better Migration Management einen konkreten Beitrag der deutschen Entwicklungszusammenarbeit zum Bereich Migrationsmanagement. Demetrios G. Papademetriou und Kate Hooper (Kap. 6) untersuchen die Entwicklung des Partnerschaftsansatzes – der Kooperation von Ziel‑, Herkunfts- und Transitländern – in der internationalen Migrationszusammenarbeit anhand von Fallbeispielen aus der europäischen Nachbarschaft sowie Nord- und Mittelamerika. Die von den Vereinigten Staaten und den Philippinen gemeinsam initiierte Initiative Migrants in Countries in Crisis (MICIC), die auf den Schutz von Migrant*innen abzielt, die von einem gewaltsamen Konflikt oder einer Naturkatastrophe betroffen sind, ist Gegenstand des Beitrages von Lukas Gehrke und Albert Kraler (Kap. 7). Kathleen Newland und Andrea Riester (Kap. 8) befassen sich mit der Diskrepanz zwischen einem Mangel an legalen Einreisemöglichkeiten für Geringqualifizierte und dem hohen Arbeitskräftebedarf im Niedriglohnsektor. Anhand des Global Compact for Safe, Orderly, and Regular Migration evaluieren sie die Wirkung von Instrumenten zur Steuerung der Einwanderung von Geringqualifizierten. Jason Gagnon und David Khoudour-Castéras (Kap. 9) geben einen Überblick über die Entwicklung der Migrationspolitik von Zielländern und analysieren Kooperationsmöglichkeiten zwischen Herkunfts- und Zielländern hinsichtlich einer symmetrischen Kosten- und Nutzenverteilung.

Mit dem Untersuchungsgegenstand gemeindebasierter Patenschaftsprogramme erreicht der Weg von Geflüchteten und Migrant*innen den wichtigen Abschnitt einer erfolgreichen Aufnahme und Integration in den Zielländern. Dabei verschieben Jennifer Bond und Gregory A. Maniatis (Kap. 10) die Perspektive von der Makro- auf die Meso- und Mikroebene. Dennis Dijkzeul und Annalisa Addis (Kap. 11) zeigen, dass humanitäre Hilfe und Entwicklungshilfe als zentrale Instrumente der Flüchtlingshilfe nicht immer nahtlos ineinandergreifen. Die Autor*innen evaluieren mit *Linking Relief, Rehabilitation and Development* (LRRD), humanitärer Übergangshilfe und Resilienz drei Ansätze hinsichtlich ihres Potenzials, die Lücke zwischen humanitärer Hilfe und Entwicklungshilfe zu schließen. Marlen de la Chaux (Kap. 12) befasst sich mit institutionellen Mängeln von Flüchtlingslagern und zeigt, wie unternehmerisches Handeln von Geflüchteten in den Lagern die institutionellen Lücken schließen kann

Christian Jetzlsperger (Kap. 13) widmet sich der Rolle der Außenpolitik bei der Bekämpfung von Fluchtursachen. Er betont dabei die Bedeutung einer politischen Strategie, die außen-, sicherheits- und entwicklungspolitische Maßnahmen vereint. Michelle Ndiaye (Kap. 14) fokussiert die Wanderungsbewegungen von Afrika nach Europa und zeigt Verbesserungspotenziale in der migrationspolitischen Zusammenarbeit der Afrikanischen Union und der EU. Fragile Staatlichkeit als Ursache für Flucht und Migration steht im Mittelpunkt des Beitrags von Jörn Grävingholt (Kap. 15). Entwicklungszusammenarbeit muss fragile Staatlichkeit dem Autor zufolge verstärkt adressieren und dafür das Konzept der Governanceförderung nachjustieren. Abschließend beschreibt Koko Warner (Kap. 16) Unterschiede zwischen politischen Ansätzen verschiedener Politikfelder im Umgang mit Themen wie Migration, Vertreibung und geplanter Umsiedlung. Dafür stellt die Autorin die Konzepte Risikomanagement und Resilienz aus dem Bereich Klimapolitik dem Management und Schutz von nationalen Grenzen im Sinne der allgemeinen Migrations- und Flüchtlingspolitik gegenüber.

Der Sammelband zeigt vor allem, dass die mit globalen Wanderungsbewegungen verbundenen Herausforderungen nicht allein durch Mittel der (inneren) Ordnungs- und Migrationspolitik gelöst werden können, sondern ein Zusammenwirken verschiedener Akteure und Politikfelder erfordern, wobei der Entwicklungszusammenarbeit eine besondere Rolle zukommt. Aufgrund der Provenienz der Autor*innen – die für Einrichtungen wie die GIZ, die Internationale Arbeitsorganisation oder das Deutsche Institut für Entwicklungspolitik (DIE) im Einsatz sind – bleiben die Beiträge nicht auf einer theoretischen Ebene. Vielmehr zeigen sie, was in der Praxis bereits umgesetzt wird und an welchen Stellen noch Handlungsbedarf besteht. Der Wert des Sammelbandes zeigt sich nicht zuletzt darin, dass die Herausgeber Ende 2021 eine englische Version des Werkes unter dem Titel *Forced Displacement and Migration* veröffentlicht haben.

